# Multi-agent modeling and simulation of *Plasmodium falciparum* dynamics considering sulfadoxine-pyrimethamine resistance and environmental factors

**DOI:** 10.1186/1475-2875-13-S1-O42

**Published:** 2014-09-22

**Authors:** Daniela Zaffalon Gomez, Andrés Olarte Dussan, Vladimir Corredor Espinel, Hernando Diaz Morales

**Affiliations:** 1 Department of Electrical and Electronics Engineering, Universidad Nacional de Colombia, Bogotá, Colombia; 2 Parasitology Laboratory, Department of Public Health, Faculty of Medicine, Universidad Nacional de Colombia, Bogotá, Colombia

## 

The appearance of resistant *Plasmodium falciparum* strains after the application of medication in human populations has generated global concern about the correct use of anti-malaria drugs. One of the challenges in malaria research is to find the correct methods to eradicate current strains while avoiding the appearance of new resistant strains. To recommend methods for malaria control, it is necessary to understand disease transmission and parasite dynamics and take into consideration spatial, temporal and environmental variations. In order to describe these elements, mathematical, stochastic and computational models have been developed.

In this work, an agent-based model represents the malaria transmission, in which the defined agents are humans and mosquitoes. The interaction between these two agents is simulated in a spatial setting considering environmental and temporal variations. In particular, the parasite is modeled as a characteristic of both agents, considering mutation and recombination and their effects on drug resistance emergence.

On the one hand, the model recreates the gonotrophic cycle of mosquito (*Anopheles gambiae*) and includes population births and deaths. It also represents the mosquito’s epidemiological states: susceptible, exposed and infected (SEI). On the other hand, the model includes a human population in which sulfadoxine-pyrimethamine is applied as an anti-malarial drug with 100% coverage. Human dynamics also includes births, deaths and migration. Additionally, the represented human epidemiological states in the model are SEIS, in which the reinfection probability depends on the disease exposure history. The interaction between both mosquitoes and humans occurs in a simulated scenario of one square kilometer including environmental conditions that affect the mosquito life cycle.

The parasite life cycle is modeled during the interaction between mosquitoes and humans. Due to selection pressure as a consequence of sulfadoxine-pyrimethamine use, strains with fixed resistant mutations at the *DHFR* and *DHPS* loci emerge. This is modeled through the emergence of fifteen drug-resistant mutations and the subsequent recombination among alleles, generating mutational pathways developing into more resistant strains. Moreover, super-infection in humans (two genotypes) and mosquitoes (two genotypes - infection and the result of recombination) is represented.

Simulations for high transmission parameters are consistent with literature, presenting coherent times for each strain appearance, and showing a mutational pathway under high transmission settings and 100% of drug coverage. Thus, measuring the prevalence of each genotype in the model allows the analysis of different cases to eradicate the disease by teh correct usage of control strategies. Furthermore, the model is being adapted for representing low transmission scenarios.

